# Factors associated with delayed initiation of breastfeeding: a cross-sectional study in South Sudan

**DOI:** 10.1186/s13006-018-0170-0

**Published:** 2018-07-05

**Authors:** Justin Bruno Tongun, Mohammed Boy Sebit, David Mukunya, Grace Ndeezi, Victoria Nankabirwa, Thorkild Tylleskar, James K. Tumwine

**Affiliations:** 1 Centre for International Health, University of Bergen, Bergen, Norway; 2 Department of Paediatrics and Child Health, College of Medicine, University of Juba, Juba, South Sudan; 3 Department of Internal Medicine, College of Medicine, University of Juba, Juba, South Sudan; 4 Department of Paediatrics and Child Health, School of Medicine, College of Health Sciences, Makerere University, Kampala, Uganda; 5 School of Public Health, College of Health Sciences, Makerere University, Kampala, Uganda

**Keywords:** Breastfeeding, Early initiation, Infant, Associated factors, South Sudan

## Abstract

**Background:**

The global breastfeeding recommendation states that all infants should be put to the breast within one hour of birth, which is defined as timely initiation or early initiation of breastfeeding. Early initiation of breastfeeding is associated with reduced risk in infant illness and death. Understanding the determinants of delay in initiation of breastfeeding might spur health staff and policy makers to foster timely breastfeeding. We assessed the prevalence and determinants of delay in initiation of breastfeeding among mothers in Juba Teaching Hospital.

**Methods:**

The present study enrolled 806 mother-infant pairs within 24 hrs of birth in Juba Teaching Hospital in 2017. The mothers were interviewed about the time of initiation of breastfeeding, sociodemographic and birth characteristics. The independent variables associated with delay in initiation of breastfeeding were identified using multivariable logistic regression analysis.

**Results:**

In the current study, 52% (418/806) of the mothers initiated breastfeeding later than one hour after birth. Birth by Caesarean section (Adjusted Odds Ratio [AOR] 41; 95% Confidence Interval [CI] 12.21, 138), discarding of colostrum (AOR 9.89; 95% CI 4.14, 23.62), unmarried mothers (AOR 3.76; 95% CI 1.53, 9.24), exposure to infant formula advertisement (AOR 1.82; 95% CI 1.09, 3.02) and no house ownership (AOR 1.52; 95% CI 1.11, 2.09) were independent factors associated with delay in initiation of breastfeeding.

**Conclusion:**

We found that more than half of the mothers delayed the initiation of breastfeeding. Therefore, we recommend training on best breastfeeding practices and counselling skills for health staff in Juba Teaching Hospital. Policy dialogue, with the relevant ministries and departments on the promotion and protection of early initiation of breastfeeding is crucial.

## Background

The universally accepted breastfeeding recommendations state that all newborn babies should be placed in skin-to-skin contact with their mothers immediately after birth and initiate breastfeeding within one hour of birth [1]. It also states that all infants should be exclusively breastfed (EBF) for six months. From six months of age introduce the child to timely, adequate, safe and appropriate complementary foods while continuing breastfeeding up to two years or beyond [2, 3].

In a recent study, 58% of infants were put to the breast within one hour of birth [4]. Early initiation of breastfeeding (EIBF) promotes the release of oxytocin that enables contraction of the uterus and decreases postpartum haemorrhage [5]. Early breast suckling facilitates production of the first breastmilk, called colostrum which has nutrients and immunity substances that protect infants from infections [5]. Evidence from studies found that EIBF was associated with a lower risk of diarrhoea in infants in Nigeria [6], reduced infant morbidity in Nepal, Vietnam and India [7–9] and reduced infant mortality in Ghana [10, 11]. However, suboptimal breastfeeding was one of the three leading causes of infant diseases in sub-Saharan Africa [12]. Delay in the initiation of breastfeeding was responsible for increased risk in infant morbidity and mortality in Ghana, India and Tanzania [13]. This practice was associated with antenatal care visits [14], birth place [15], home area, household income [16, 17], prelacteal feeding [18], birth by Caesarian section (CS) and colostrum discarding [19].

The present study was carried out in South Sudan, a country plagued by wars since the 1950s and only punctuated by peace, in 2005 to 2013. These conflicts traumatized women and children, consequently influencing health seeking behaviour and infant feeding. In 2016, the United Nations Children’s Fund (UNICEF) estimated that 48% of infants were breastfed early; while 45% were exclusively breastfed for six months [20]. There is little known about the determinants of early breastfeeding practices in South Sudan. This, Africa’s youngest nation is fast introducing a number of WHO/UNICEF promoted policies and practices. However, it is not clear to what extent these policies have been implemented. For example, there is a draft policy on infant and young child feeding which apparently, incorporated the Baby Friendly Hospital Initiative (BFHI). To date, however no hospital in the country has yet started implementing the BFHI policy. Therefore, identifying predictors of delay in initiation of breastfeeding is critical in designing strategies to improve early initiation of breastfeeding. This study sought to establish factors associated with delay in initiation of breastfeeding in South Sudan.

## Methods

### Study setting

This study was conducted at the maternity ward in Juba Teaching Hospital (JTH) in South Sudan. This is a national referral and teaching hospital for the College of Medicine, University of Juba. The hospital has a capacity of 500 beds and conducts, on average, 20 births daily.

### Study design and sample

This was a cross-sectional study among 806 mother-infant pairs conducted from October 2016 to January 2017. Mothers who gave informed written consent, had a term birth and live infant were included. We excluded mothers who had ill infants; and those unable to communicate independently.

### Sample size estimation

This study considered a 50% prevalence of early initiation of breastfeeding in sub-Saharan Africa from a recent descriptive analysis in 57 low-and middle-income counties [21], with a precision of 5%, 95% confidence intervals and non-response of 20%. This gave us sample size of 461 participants. This sample size was determined using Open Epi [22].

We also calculated a sample size for factors associated with delay in initiation of breastfeeding. Initially, the sample sizes of various exposures were computed using Open-Epi [22] epidemiological calculator for detecting differences between proportions of two groups (Kelsey formula). Finally, discarding of colostrum was used to calculate the sample size needed for detecting variations between proportions of delay in initiation of breastfeeding between mothers who discarded colostrum and those who did not. Using a study from the Somali region of Ethiopia where 46.3% of the mothers discarded colostrum, while 53.7% did not [23], we assumed the proportion of delay in initiation of breastfeeding of 59% for mothers who discarded colostrum and 49% for those who have not discarded. This gave a sample size of 806; which was enough to detect differences in the proportions of the exposures of interest with 80% power.

### Sampling procedure

**Fig. 1:**
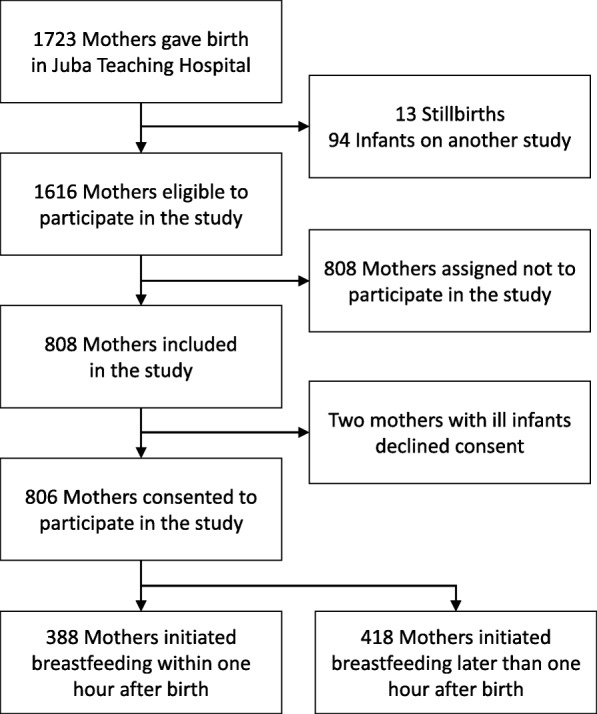
Flowchart of mother-infant pairs in Juba Teaching Hospital, South Sudan

A total of 1723 mothers gave birth in JTH, of these 13 had stillbirths; and 94 mothers were enrolled in another study. The study objectives and procedures were explained to the 1616 mothers eligible to participate in the study. In the design of the study, we planned to recruit 10 of the 20 mothers who gave birth daily. However, consecutive sampling would have favored those mothers who gave birth in the day and left out those who gave birth at night; who may have different characteristics. It was also not practical to randomly sample 10 mothers after all births had occurred in a day, because most mothers leave the hospital few hours after birth. Therefore, we decided to randomly sample one of every two mothers who gave birth. This process spread the data collection throughout the day. So, for every two births, the mothers were requested to pick a concealed piece of paper from a box. Those who picked a “yes” were assigned to “participate” and those picking the “no” option to “not to participate” in the study (Fig. 1). Of the 808 mothers who picked to participate in the study, 806 consented to participate in the study; while two mothers with ill infants declined consent.

### Data collection and instruments

We used a questionnaire generated from the World Health Organization (WHO) indicators for assessing infant and young child feeding practices [24] and a study in Uganda [25]**.** The questionnaire was translated to *Bari* (the local language) and back translated into English by a different translator for validation. Next, it was piloted and pretested in Al Sabbah Children Hospital in Juba. After the pilot test, irrelevant questions were removed, vague questions made clearer and errors corrected. The questionnaire had three sections: sociodemographic characteristics, birth characteristics, and breastfeeding practices. The data were collected by a pair of trained research assistants in a face to face interview. The collected data were checked daily for completeness, consistency and errors by the principal investigator (PI). The data were cleaned, coded, double entered into Epi Info version 6 and stored safely in a password-protected computer to which the PI had sole access.

### Study variables

The outcome variable was timely or early initiation of breastfeeding. The mothers were asked how long after birth their babies were first put to the breast. Initiating breastfeeding within one hour was categorized as “early”, whereas later than one hour was categorized as “late /delayed”. The independent variables have three sections:

1. *Sociodemographic characteristics* including maternal age, marital status, parity, place of residence, educational status, employment status, religion, household assets, house ownership, age of infant, and infant sex.
2. *Maternal health services* including antenatal care, place of birth, assistance during birth, mode of delivery, type of birth, birth order, breastfeeding counseling, breastfeeding support; and exposure to infant formula advertisement through radio, TV or newspapers one month before birth.
3. *Breastfeeding practices* including prelacteal feeding and colostrum discarding.

### Data processing and analysis

In the present study, we reported the descriptive variables as means and standard deviations. The categorical variables were reported as proportions. Chi square tests were performed to identify the independent variables that were associated with outcome variable of interest and recorded their *p* - values. Factors from the literature known to predict our outcome variable and those with a *p* - value ≤0.25 at binary analysis, not in the causal pathway and not strongly collinear with other independent variables were entered into the initial multivariable logistic regression model. We evaluated for collinearity and any factor with variance inflation factor (VIF) > 10 was regarded as strongly collinear. In instances where there was collinearity, the factor with a stronger measure of association with the outcome variable was retained in the model and the other dropped. The variables with a *p* - value ≤0.25 from bivariate analysis were then entered into a backward stepwise multivariate model. Next interaction terms were formed between the variables retained in the multivariate model. The reduced model was then compared with the model with the interaction terms using chunk test to assess for interaction. We evaluated the confounding effect for the variables dropped from the model on the variables that remained in the multivariate model. Any variable that caused a variation ≥10% between the crude and adjusted degrees of association of any of the variables that remained in the multivariate model was kept in the model as a confounder. In the final model, independent variables with a *p* - value < 0.05 were regarded key associated factors. Finally, we carried out the multivariable logistic regression analysis without discarding of colostrum. Since this variable could be a consequence and not a cause of the delay in initiation of breastfeeding. For example, discarding of colostrum could result from delay in initiation of breastfeeding due to mother’s engorged breast or infant illness. To control for this situation, we repeated the analysis without this variable. Data analysis for the current study was conducted using STATA version 14 (Stata Corp LLC, Texas, USA).

## Results

### Baseline and birth characteristics

The mean age of the mothers was 24.9 years (SD 5.3) (Tables 1 and 2). Of all the mothers, more than half 51% lived in urban area, 46% owned houses, 96% were married, 17% were uneducated, and 76% were unemployed. The majority, 91% of the mothers attended antenatal care (ANC), 88% gave birth normally, 55% received breastfeeding counseling, and 88% were not exposed to infant formula advertisement a month before birth.

**Table 1:** Baseline characteristics of mother-infant pairs and delayed initiation of breastfeeding in Juba Teaching Hospital, South Sudan

	All participants *n* = 806	Delayed initiation^a^ of breastfeeding *n* = 418
Variables	*n* (%)	*n* (%)
Infant sex		
Male	386 (47.9)	209 (50.0)
Female	420 (52.1)	209 (50.0)
Mother age		
≤ 19	139 (17.2)	73 (17.5)
20–24	252 (31.3)	126 (30.1)
25–29	246 (30.5)	135 (32.3)
30–34	123 (15.3)	57 (13.6)
≥ 35	46 (5.7)	27 (6.5)
Place of residence		
Urban	410 (50.9)	204 (48.8)
Rural	396 (49.1)	214 (51.2)
House ownership		
Yes	373 (46.3)	167 (40.0)
No	433 (53.7)	251 (60.0)
Mother marital status		
Married	775 (96.2)	394 (94.3)
Single	31 (3.8)	24 (5.7)
Mother education status		
None	136 (16.9)	65 (15.6)
Primary	377 (46.8)	194 (46.4)
Secondary	239 (29.7)	128 (30.6)
Tertiary	54 (6.7)	31 (7.4)
Mother employment status		
Employed	197 (24.4)	101 (24.2)
Not employed	609 (75.6)	317 (75.8)

^a^Delayed initiation refers to putting an infant to the breast later than 1 hr after birth

**Table 2:** Birth characteristics of mother-infant pairs and delayed initiation of breastfeeding in Juba Teaching Hospital, South Sudan

	All participants *n* = 806	Delayed initiation^a^ of breastfeeding *n* = 418
Variables	*n* (%)	*n* (%)
Antenatal care visit		
2 or more	731 (90.7)	368 (88.0)
0–1	75 (9.3)	50 (12.0)
Skilled birth attendant		
Yes	788 (97.8)	406 (97.1)
No	18 (2.2)	12 (2.9)
Mode of birth		
Normal vaginal	709 (88.0)	324 (77.5)
Caesarean section	97 (12.0)	94 (22.5)
Birth type		
Single	790 (98.0)	407 (97.4)
Multiple	16 (2.0)	11 (2.6)
Birth order		
Primipara	523 (64.9)	263 (62.9)
Multipara	283 (35.1)	155 (37.1)
Exposure to infant formula advertisement one month before birth		
No	711 (88.2)	354 (84.7)
Yes	95 (11.8)	64 (15.3)
Breastfeeding counseling		
Yes	445 (55.2)	241 (57.7)
No	361 (44.8)	177 (42.3)
Breastfeeding support		
Yes	459 (56.9)	232 (55.5)
No	347 (43.1)	186 (44.5)
Discarding of colostrum		
No	739 (91.7)	357 (85.4)
Yes	67 (8.3)	61 (14.6)

^a^Delayed initiation refers to putting an infant to the breast later than 1 h after birth

### Bivariate analysis

More than half 52% (418/806) of the mothers delayed initiation of breastfeeding. Place of residence, marital status, no house ownership, ANC, mode of birth, birth order, type of birth, discarding of colostrum, and exposure to infant formula advertisement were associated with delay in initiation of breastfeeding (Table 3). The variables which were not associated at this level were excluded in final multivariable analysis.

**Table 3:** Bivariate and multivariable analysis for delayed in initiation of breastfeeding in Juba Teaching Hospital, South Sudan

	Bivariate *n* = 806	Multivariable *n* = 806
Variables	OR (95% CI)	AOR^a^ (95% CI)
Infant’s sex		
Male	1	
Female	0.84 (0.64, 1.11)	–
Mother’s age		
≤ 19	1	
20–24	0.90 (0.60, 1.37)	–
25–29	1.10 (0.72, 1.67)	–
30–34	0.78 (0.48, 1.27)	–
≥ 35	1.28 (0.65, 2.52)	–
Place of residence		
Urban	1	
Rural	1.19 (0.90, 1.57)	–
House ownership		
Yes	1	1
No	1.70 (1.29, 2.45)	1.52 (1.11, 2.09)
Mother marital status		
Married	1	1
Unmarried	3.32 (1.41, 7.79)	3.76 (1.53, 9.24)
Mother education status		
None	1	
Primary	1.56 (0.78, 1.71)	–
Secondary	1.26 (0.83, 1.92)	–
Tertiary	1.47 (0.36, 1.28)	–
Mother employment status		
Employed	1	
Not employed	1.03 (0.75, 1.42)	–
Antenatal care visits		
2 or more	1	
0–1	1.97 (1.19, 3.26)	1.53(0.87, 2.71)
Skilled birth attendant		
Yes	1	
No	1.88 (0.70, 5.06)	–
Mode of birth		
Normal vaginal	1	
Caesarean section	37.23 (11.68, 119)	41 (12.21, 138)
Birth type		
Single	1	
Multiple	2.07 (0.71, 6.01)	–
Birth order		
Multipara	1	
Primipara	1.20 (0.90, 1.60)	1.22 (0.88, 1.68)
Exposure to infant formula advertisement one month before birth		
No	1	1
Yes	2.08 (1.32, 3.28)	1.82 (1.09, 3.02)
Breastfeeding counseling		
Yes	1	
No	0.81(0.62, 1.08)	–
Breastfeeding support		
Yes	1	
No	1.13 (0.85, 1.49)	–
Discarding of colostrum		
No		
Yes	10.88(4.65, 25.47)	9.89 (4.14, 23.62)

^a^*AOR* adjusted odds ratio

### Multivariable analysis

In this analysis, birth by Caesarean section (Adjusted odd ratio [AOR] 41; 95% confidence interval [CI] 12.21, 138), discarding of colostrum (AOR 9.89; 95% CI 4.14, 23.62), unmarried mothers (AOR 3.76; 95% CI 1.53, 9.24), exposure to infant formula advertisement (AOR 1.82; 95% CI 1.09, 3.02), and no house ownership (AOR 1.52; 95% CI 1.11, 2.09) were associated with delay in initiation of breastfeeding (Table 3). The repeated model without discarding of colostrum found the same results with similar measure of association.

## Discussion

In this study, more than half of the mothers initiated breastfeeding later than one hour after birth. Factors associated with this practice were birth by Caesarean section, discarding of colostrum, unmarried mothers, no house ownership and exposure to infant formula advertisement.

According to the WHO rating on early initiation of breastfeeding; 0–29% is considered poor, 30–49% as fair, 50–89% as good and 90–100% as very good [26]. The prevalence of early initiation of breastfeeding in the present study is fair. This was similar to that of a study in Ethiopia [27]. This was however, lower than that of other studies in Mulago hospital (69%) [16], rural Uganda (57%) [28], and Ethiopia (84%) [29].

There was strong evidence that birth by Caesarean section (CS) was associated with delay in initiation of breastfeeding. This was similar to findings from Uganda [30], Tanzania [31], Ethiopia [27], and Nigeria [32]. This could be explained by the hospital practice of separating infants from their mothers after CS as reported in studies in Nigeria [33] and Vietnam [34]. This also could be due to fatigue and pain experienced by the mother after birth [16].

Mothers who were exposed to infant formula advertisement were two times more likely to delay initiation of breastfeeding. This was similar to that of a study in Cambodia [35]. A recent systematic review found that infant formula promotion undermined breastfeeding, urged mothers to stop breastfeeding and gave prelacteal feeding [36]. A study in Nepal found that promotion of infant formula by health staff contributed to suboptimal breastfeeding [37].

Discarding of colostrum was strongly associated with delay in initiation of breastfeeding. However, discarding colostrum could be a consequence and not a cause of delay in initiation of breastfeeding. To counter this situation, the multivariable analysis was repeated without discarding of colostrum, but the findings were similar. In this study, mothers thought colostrum was dirty and bad. This finding was similar to studies in Guinea-Bissau [38], Ethiopia [39] and Guatemala [40]. Negative cultural beliefs were responsible for discarding of colostrum. However, our study did not explore the effect of cultural beliefs on discarding of colostrum.

Unmarried mothers were four times more likely to practice delay in initiation of breastfeeding compared to married mothers. This was similar to recent findings in the Democratic Republic of Congo [41] and Tanzania [31]. Another study in the USA reported delayed initiation of breastfeeding among unmarried mothers who gave birth to preterm infants [42]. The present study however, did not investigate the reasons underlying delay in initiation of breastfeeding among the unmarried mothers.

Lastly, mothers who did not own houses were two times more likely to breastfeed later than one hour after birth compared to those who own houses. This was similar to that of a study in Ethiopia [29]. The mothers who breastfed later than one hour after birth may most likely be women living in poverty and were given less opportunity to initiate breastfeeding early.

### Strengths and limitations

This study was the first to assess early initiation of breastfeeding in a unique conflict setting in South Sudan. The data were gathered within 24 hrs after birth which minimized recall bias. This study had several limitations; the result of this study cannot be generalized since one hospital was studied. This study omitted other methods of infant formula advertisement such as points of sale, social media promotion, internet, health staff role and public display. In addition, the information on infant formula advertisement could be subject to recall bias. Finally, the mothers’ ability to estimate exact time on initiation of breastfeeding was not assessed.

## Conclusion

In this study, delay in initiation of breastfeeding was prevalent in Juba Teaching Hospital. Birth by Caesarean section, discarding of colostrum, exposure to infant formula advertisement, unmarried mothers and no house ownership were associated with delay in initiation of breastfeeding. We recommend health staff training on best breastfeeding practices and counselling skills, especially the staff responsible for birth by Caesarean section. In-depth study on cultural beliefs and socioeconomic determinants with special focus on discarding of colostrum and house ownership is urgent. Policy dialogue is crucial with the Ministry of Health to review compliance with WHO policies, especially the Code of Marketing Breast Milk Substitutes. Initiation of the BFHI might go a long way in mitigating the challenge of delay in initiation of breastfeeding in the hospital. Lastly, a qualitative study is recommended to investigate the rational for delay in initiation of breastfeeding among unmarried mothers.

## Abbreviations

ANC: Antenatal care
CS: Caesarean section
EBF: Exclusive breastfeeding
JTH: Juba Teaching Hospital
VIF: Variance inflation factor
WHO: World Health Organization

## References

[1] World Health Organization. Guideline: protecting, promoting and supporting breastfeeding in facilities providing maternity and newborn services. Geneva: World Health Organization; 2017. Available from: http://apps.who.int/iris/handle/10665/259386. Accessed 18 May 2018. 29565522

[2] World Health Organization. Global strategy for infant and young child feeding. Geneva: World Health Organization; 2003. Available from: http://www.who.int/nutrition/publications/infantfeeding/9241562218/en/. Accessed 18 May 2018.

[3] Victora CG, Bahl R, Barros AJ, Franca GV, Horton S, Krasevec J (2016). Breastfeeding in the 21st century: epidemiology, mechanisms, and lifelong effect. Lancet.

[4] Takahashi K, Ganchimeg T, Ota E, Vogel JP, Souza JP, Laopaiboon M (2017). Prevalence of early initiation of breastfeeding and determinants of delayed initiation of breastfeeding: secondary analysis of the WHO global survey. Sci Rep.

[5] Palmeira P, Carneiro-Sampaio M (2016). Immunology of breast milk. Rev Assoc Med Bras.

[6] Ogbo FA, Page A, Idoko J, Claudio F, Agho KE (2016). Diarrhoea and suboptimal feeding practices in Nigeria: evidence from the national household surveys. Paediatr Perinat Epidemiol.

[7] Mullany LC, Katz J, Li YM, Khatry SK, LeClerq SC, Tielsch JM (2008). Breast-feeding patterns, time to initiation, and mortality risk among newborns in southern Nepal. J Nutr.

[8] Hajeebhoy N, Nguyen PH, Mannava P, Nguyen TT, Mai LT (2014). Suboptimal breastfeeding practices are associated with infant illness in Vietnam. Int Breastfeed J.

[9] Garcia CR, Mullany LC, Rahmathullah L, Katz J, Thulasiraj RD, Tielsch JM (2011). Breastfeeding initiation time and neonatal mortality risk among newborns in South India. J Perinatol.

[10] Edmond KM, Kirkwood BR, Amenga-Etego S, Owusu-Agyei S, Hurt LS (2007). Effect of early infant feeding practices on infection-specific neonatal mortality: an investigation of the causal links with observational data from rural Ghana. Am J Clin Nutr.

[11] Edmond KM, Kirkwood BR, Tawiah CA, Owusu-Agyei S (2008). Impact of early infant feeding practices on mortality in low birth weight infants from rural Ghana. J Perinatol.

[12] Lim SS, Vos T, Flaxman AD, Danaei G, Shibuya K, Memish ZA (2012). A comparative risk assessment of burden of disease and injury attributable to 67 risk factors and risk factor clusters in 21 regions, 1990-2010: a systematic analysis for the global burden of disease study 2010. The Lancet.

[13] Edmon K, Newton S, Hurt L, Shannon SG, Mazumder S, Taneja S (2016). Timing of initiation, patterns of breastfeeding, and infant survival: prospective analysis of pooled data from three randomised trials. Lancet.

[14] Ekubay M, Berhe A, Yisma E (2018). Initiation of breastfeeding within one hour of birth among mothers with infants younger than or equal to 6 months of age attending public health institutions in Addis Ababa, Ethiopia. Int Breastfeed J.

[15] Yimer NB, Liben ML (2018). Effects of home delivery on colostrum avoidance practices in north Wollo zone, an urban setting, Ethiopia: a cross sectional study. Matern Child Nutr.

[16] Kalisa R, Malande O, Nankunda J, Tumwine JK (2015). Magnitude and factors associated with delayed initiation of breastfeeding among mothers who deliver in Mulago hospital. Uganda African Health Sciences.

[17] Engebretsen IM, Nankabirwa V, Doherty T, Diallo AH, Nankunda J, Fadnes LT (2014). Early infant feeding practices in three African countries: the PROMISE-EBF trial promoting exclusive breastfeeding by peer counsellors. Int Breastfeed J.

[18] Legesse M, Demena M, Mesfin F, Haile D (2014). Prelacteal feeding practices and associated factors among mothers of children aged less than 24 months in Raya kobo district, north eastern Ethiopia: a cross-sectional study. Int Breastfeed J.

[19] Tilahun G, Degu G, Azale T, Tigabu A (2016). Prevalence and associated factors of timely initiation of breastfeeding among mothers at Debre Berhan town, Ethiopia: a cross-sectional study. Int Breastfeed J.

[20] UNICEF. The state of the world's children. New York: UNICEF; 2016. Available from: http://www.unicef.org/sowc2014/numbers. Accessed 4 January 2018.

[21] Oakley L, Benova L, Macleod D, Lynch CA, Campbell OMR (2018). Early breastfeeding practices: descriptive analysis of recent demographic and health surveys. Maternal & Child Nutrition..

[22] Dean AG, Sullivan KM, Soe MM. Open epi: open source epidemiologic statistics for Public Health; 2013. Available from: www.OpenEpi.com. Accessed 4 September 2017

[23] Rogers NL, Abdi J, Moore D, Nd'iangui S, Smith LJ, Carlson D (2011). Colostrum avoidance, prelacteal feeding and late breast-feeding initiation in rural northern Ethiopia. Public Health Nutr.

[24] World Health Organization. Indicators for assessing infant and young child feeding practices. Geneva: World Health Organization; 2010. Available from: http://www.who.int/nutrition/publications/infantfeeding/9789241599290/en/. Accessed 19 May 2018.

[25] Tylleskar T, Jackson D, Meda N, Engebretsen IM, Chopra M, Diallo AH (2011). Exclusive breastfeeding promotion by peer counsellors in sub-Saharan Africa (PROMISE-EBF): a cluster-randomised trial. Lancet.

[26] World Health Organization. Infant and young child feeding: a tool for assessing national practices, policies and programmes. Geneva: World Health Organization; 2003. Available from: http://www.who.int/iris/handle/10665/42794. Accessed 10 May 2018.

[27] Liben ML, Yesuf EM (2016). Determinants of early initiation of breastfeeding in Amibara district, northeastern Ethiopia: a community based cross-sectional study. Int Breastfeed J.

[28] Waiswa P, Peterson S, Tomson G, Pariyo GW (2010). Poor newborn care practices - a population based survey in eastern Uganda. BMC Pregnancy and Childbirth.

[29] Beyene MG, Geda NR, Habtewold TD, Assen ZM. Early initiation of breastfeeding among mothers of children under the age of 24 months in southern Ethiopia. Int Breastfeed J. 2017;12:1. 10.1186/s13006-016-0096-3 PMC5217403 28070207

[30] Mukunya D, Tumwine JK, Nankabirwa V, Ndeezi G, Odongo I, Tylleskar T (2017). Factors associated with delayed initiation of breastfeeding: a survey in northern Uganda. Glob Health Action.

[31] Exavery A, Kante AM, Hingora A, Phillips JF (2015). Determinants of early initiation of breastfeeding in rural Tanzania. Int Breastfeed J.

[32] Berde AS, Yalcin SS (2016). Determinants of early initiation of breastfeeding in Nigeria: a population-based study using the 2013 demographic and health survey data. BMC Pregnancy Childbirth.

[33] Awi DD, Alikor EA (2006). Barriers to timely initiation of breastfeeding among mothers of healthy full-term babies who deliver at the University of Port Harcourt Teaching Hospital. Niger J Clin Pract.

[34] Bui QT, Lee HY, Le AT, Van Dung D, Vu LT (2016). Trends and determinants for early initiation of and exclusive breastfeeding under six months in Vietnam: results from the multiple indicator cluster surveys, 2000-2011. Glob Health Action.

[35] Pries AM, Huffman SL, Mengkheang K, Kroeun H, Champeny M, Roberts M (2016). Pervasive promotion of breastmilk substitutes in Phnom Penh, Cambodia, and high usage by mothers for infant and young child feeding. Maternal & Child Nutrition.

[36] Rollins NC, Bhandari N, Hajeebhoy N, Horton S, Lutter CK, Martines JC (2016). Why invest, and what it will take to improve breastfeeding practices?. Lancet.

[37] Pries AM, Huffman SL, Adhikary I, Upreti SR, Dhungel S, Champeny M (2016). Promotion and prelacteal feeding of breastmilk substitutes among mothers in Kathmandu valley. Nepal Maternal & Child Nutrition.

[38] Gunnlaugsson G, da Silva MC, Smedman L (1992). Determinants of delayed initiation of breastfeeding: a community and hospital study from Guinea-Bissau. Int J Epidemiol.

[39] Hailemariam TW, Adeba E, Sufa A (2015). Predictors of early breastfeeding initiation among mothers of children under 24 months of age in rural part of West Ethiopia. BMC Public Health.

[40] Atyeo NN, Frank TD, Vail EF, Sperduto WAL, Boyd DL (2017). Early initiation of breastfeeding among maya mothers in the western highlands of Guatemala: practices and beliefs. J Hum Lact.

[41] Kambale RM, Buliga JB, Isia NF, Muhimuzi AN, Battisti O, Mungo BM (2018). Delayed initiation of breastfeeding in Bukavu, south Kivu, eastern Democratic Republic of the Congo: a cross-sectional study. Int Breastfeed J.

[42] Kair LR, Colaizy TT (2016). Breastfeeding continuation among late preterm infants: barriers, facilitators, and any association with neonatal intensive care unit admission?. Hospital Pediatrics.

